# Primary Health Care Case-Management Nurses during the COVID-19 Pandemic: A Qualitative Study

**DOI:** 10.3390/nursrep14020084

**Published:** 2024-04-28

**Authors:** María José Molina-Gil, María Dolores Guerra-Martín, Rocío De Diego-Cordero

**Affiliations:** 1 Southern Seville Health Management Area, Andalusian Health Service, 41071 Sevilla, Spain; mariajosemolinagil@hotmail.com; 2 Francisco Maldonado of Osuna University Center, 41640 Osuna, Seville, Spain; 3 Department of Nursing, Faculty of Nursing, Physiotherapy, and Podiatry, University of Seville, 41009 Seville, Spain; rdediego2@us.es; 4 Institute of Biomedicine of Seville (IBiS), 41013 Seville, Spain

**Keywords:** case management, COVID-19 pandemic, nurses, qualitative research

## Abstract

The COVID-19 has caused high morbidity and mortality in vulnerable people, such as those affected by chronic diseases, and case-management nurses (CMNs) are reference professionals for their health care and management. The objective of this study is to better understand the discourse, experiences, and feelings about the professional performance of CMNs during the pandemic. A qualitative study was conducted by conducting semi-structured interviews with CMNs (n = 31) from the province of Seville (Spain) and performing a narrative discourse analysis. The Atlas Ti 6.2 software program was used. Two categories were defined: 1. CMNs’ competencies (76 verbatim testimonies); and 2. Consequences of the COVID-19 pandemic (61 verbatim testimonies). This study was granted due permission by the Research Ethics Committee belonging to the University of Seville, under protocol code: 1139-N-22. The pandemic caused an increase in CMNs’ workload, and they had to assume their usual care tasks for vulnerable populations in addition to simultaneously prioritizing assistance in nursing homes. We can highlight CMNs’ adaptation to the pandemic situation and to these new requirements in the context of their significant social commitment to the advanced practice of the profession, a commitment that is closely related to leadership. We should also indicate that interpersonal relationships were improved, and that there was technological progress. Some CMNs mentioned an increase in their workload and reported experiencing burnout syndrome. We conclude that CMNs’ management of health care during the pandemic has been extraordinary, especially in regard to the most vulnerable populations of patients, including individuals with chronic diseases and institutionalized older adults, a fact that has been valued by the institutions and by society in general.

## 1. Introduction

The COVID-19 pandemic has especially affected the most vulnerable population groups, such as older adults and individuals with chronic diseases, causing significant morbidity and mortality [1,2]. COVID-19 can affect people with chronic diseases; however, the long-term effects are unknown [3]. In addition to respiratory problems, we highlight other risk factors such as advanced age, male gender, obesity, smoking habit, arterial hypertension, diabetes, neoplasms, liver and heart diseases, and chronic kidney disease, among others [4,5,6]. The implementation of specific protocols and more in-depth knowledge about diagnosis and treatment have allowed for a reduction in mortality rates [7]. Therefore, health systems should be improved at the global level, and health policies changed in order to attain greater efficiency for future pandemics [8].

Spain was the country with the third highest mortality rate due to COVID-19 during the pandemic. Mortality among older adults in nursing homes was higher than it was among those living in their own homes. Nurses’ work during the pandemic has been significant [9,10]. Initially known as liaison nurses and later on designated as CMNs (both names refer to the same professional profile), the figure of case-management nurses (CMNs) was implemented in the autonomous community of Andalusia (Spain), based on Decree No. 137/2002 of Support for Andalusian Families [11].

CMNs focus their performance on a standardized system for the comprehensive assessment of patients and caregivers, which includes the management of technical aids to ease home-based care and coordination (both interdisciplinary and across levels). They develop their activities in hospital care and in primary health care (PHC). PHC includes social-health care (home, health center, and nursing home) [12,13]. Among the roles or competencies inherent to CMNs, we can mention the following: advanced practice clinical nursing, complex care coordination, the management of chronic problems, professional practice and leadership, end-of-life care management, and coordination across levels [14,15]. The COVID-19 pandemic imposed a situation marked by helplessness for chronic patients; however, CMNs adapted well to this new circumstance, showing commitment and responsibility in the face of the pandemic [16,17].

Given all of the above, the objective of this study is to better understand the discourse, experiences, and feelings about the professional performance of primary health care CMNs during the COVID-19 pandemic.

The advantage of the study is focused on gaining insight into the professional activity of EGCs during the COVID-19 pandemic and assessing whether the activity carried out by these professionals can be of benefit in the face of the risk of future pandemics.

On the other hand, the study aims to illustrate the benefit of advanced practice professional profiles, which can improve care for the most vulnerable populations, such as the elderly, both at home and at the level of socio-health centers.

## 2. Materials and Methods

### 2.1. Research Design

The study was conducted following a qualitative methodology, which is ideal for learning about the CMNs’ discourse, experiences, and feelings during the COVID-19 pandemic, therefore providing in-depth information. Semi-structured interviews were conducted with the CMNs from the province of Seville [18,19]. The study follows the Standards for Reporting Qualitative Research (SRQR).

### 2.2. Participants, Sampling and Recruitment

The sample population consisted of 61 CMNs working in primary health care (PHC) in the province of Seville, which is divided into five PHC health districts (PHCDs). A total of 31 CMNs were selected by convenience sampling [20] (Table 1).

### 2.3. Data Collection

After contacting the CMNs by means of email messages or phone calls, a place, date, and time was agreed upon to carry out the in-person interviews during working hours. The interviews were semi-structured and in-depth, without time limitations, allowing the interviewees to freely express their opinions and comment on their experience. They were carried out in a scheduled manner during working hours, with the interviewer traveling to the area chosen by the interviewee, in order to conduct the interview as comfortably as possible, without affecting his or her personal and family life.

Translated with DeepL.com (free version), the interviews were conducted following a previously designed script where questions were asked about their professional performance during the COVID-19 pandemic, addressing the CMNs’ competencies regarding adaptation and changes during the pandemic and in terms of workload. In addition, the consequences of the pandemic in relation to the professional performance of CMNs were also addressed, in terms of institutional relations, relationships among the work team members, and work organization. The interviews lasted between 40 and 50 min.

### 2.4. Data Analysis

The interviews were recorded and subsequently transcribed and systematized into categories, establishing relationships between them. A narrative discourse analysis was performed by coding and categorizing the topics that emerged during the interviews.

The categorization was developed taking into account situations, contexts, activities, relationships between people, behaviors, opinions, and feelings.

The tool used to ease data analysis was the Atlas Ti 6.2. software program, in which the text was gradually segmented into quotes, easing coding, and writing notes or comments.

### 2.5. Ethical Considerations

The CMNs were previously informed about the study objective and all of them voluntarily signed the informed consent form. This study was carried out according to the Declaration of Helsinki and was approved by the Research Ethics Committee belonging to the University of Seville (Spain), under internal protocol code 1139-N-22 of 28 September 2022.

## 3. Results

### 3.1. Sociodemographic Characteristics of the Sample

The sample consisted of 31 CMNs (77% women and 23% men). Regarding the age of the CMNs included in the sample, four were between 40 and 50 years old, 18 were aged from 50 to 60 years old, and nine were over the age of 60. In relation to professional experience in their job position, 11 had 20 years, 10 had 21 years, and another 10 had 13 years of experience.

### 3.2. Analysis Categories

The analysis categories are shown in Table 2, along with the subcategories and occurrence frequency of the verbatim testimonies.

### 3.3. CMNs’ Competencies

#### 3.3.1. Workload

Most of the CMNs stated significant workloads, a new situation where each individual made their best despite the difficulties.

*The work volume drew my attention at that moment, it was a new situation that I‘d never experienced before…*

(3_SSD_62_W)

*It was overwhelming because we had no rest there, I used to work in the mornings, afternoons and nights and also during the weekends all day long… I think that we worked quite a deal, it was exhausting…*

(5_SSD_63_W)

This workload was even maintained during weekends and holidays, both via phone calls and in-person when it was necessary to go to the nursing home for any outbreak or because diagnostic tests had to be carried out.

*Exclusive dedication…always keeping an eye on the phone…, always ready each time they called us from the nursing home, total commitment*

(4_SSD_55_W)

#### 3.3.2. Nursing Homes

The CMNs especially highlight the incommensurable work done in nursing homes.

*Huge organization work in the nursing homes, managing and preventing outbreaks and in relation to their size…*

(10_SD_61_W)

Among the activities performed in the nursing homes, it was necessary to medicalize some of them.

*(…) we medicalized and we worked really hard with the professionals…*

(18_SD_58_M)

During the pandemic, CMNs took on important tasks such as contingency plans, data updates, or training of professionals, with the objective of preventing contagion and controlling these centers.

*It meant an important increase in work for me, the contingency plan issue, whether you have to include it in the platform, a lot of bureaucracy too that made things work…*

(16_SD_59_M)

*(…) we helped them run the nursing home, somewhat to plan everything*

(17_SD_55_W)

#### 3.3.3. Changes in the Competencies

Training the nursing homes’ personnel was an urgent need, although the nurses already devoted themselves to training in those settings.

*(…) we went there for specific issues and suddenly they tell us that we should train the nursing home’s workers*

(31_ESD_63_W)

On the other hand, this activity imposed by the pandemic and the needs presented by the nursing homes and the rest of the centers led CMNs to be cease devoting themselves to some activities and competencies inherent to case management, prioritizing nursing homes.

*It’s all been centralized in our case in the nursing homes, where each time there was an outbreak, which was practically every day, then I think that our work has been extremely important, the thing is that we’ve stopped doing purely case management tasks*

(12_SD_53_M)

*If we had to apply a vaccine, we applied a vaccine, the homes, following-up the contacts it was quite a thing*

(15_SD_65_W)

In the PHC centers, CMNs also performed new tasks related to the pandemic and, in many facilities, they put into practice the expert clinical leadership that characterizes this job position, taking care of collecting samples at the home level, vaccinating the dependent population at their homes, and controlling close contacts, in addition to a myriad of activities related to the pandemic.

*(…) besides, when the first COVID-positive case is discharged, we have to go to their house to take a sample… the CMN that supposedly knows best has to go…. Well, there goes the CMN to safely take the first sample from a patient that tested positive*

(31_ESD_63_W)

Among the CMNs’ competencies, collaboration with the home aid service professionals is observed during the pandemic, as well as at the association level and in social-health centers and institutions.

*As I see it, the home aid assistants have been great collaborators, they were our eyes and I‘ve felt that I‘ve helped them a lot, that I was their reference and I‘ve felt really well*

(30_ESD_50_W)

*(…) we also worked with local associations telling them what to do, how to protect themselves…*

(15_SD_65_W)

Among the changes, activities inherent to CMNs were also lost during the pandemic, such as team meetings, training sessions, and meetings or workshops with the caregivers, which meant a loss for the community.

*The pandemic made workshops and meetings to be lost, all things related to teamwork with the meetings and in the community…*

(15_SD_65_W)

*The workshops for caregivers were suspended…*

(31_ESD_63_W)

Activities inherent to CM were preserved, such as home-based assistance for patients in need of palliative care.

*(…) I kept making home visits in the middle of the pandemic, obviously fewer than before*

(12_SD_53_M)

*Case-management nurses have always been ready from the beginning of the pandemic, we haven’t stopped making our home visits, assisting the patients we had in the homes with severe health problems*

(1_ESD_62_W)

The case management tasks were still performed but care in nursing homes was gradually prioritized.

*Work was never stopped, assigning priorities because some lines had to be done no matter what, with nursing homes among them*

(23_AD_43_W)

### 3.4. Consequences of the COVID-19 Pandemic

#### 3.4.1. Technological Progress

A positive aspect to be noted is technological progress, which improved communication and accessibility. The pandemic promoted teleconsultation as a novelty that had not previously been done. This care through teleconsultation made it possible to know the health status of patients without the need to travel to their homes, in order to avoid contagion. In these teleconsultations, nursing interventions were carried out, with nurses providing information, advice, and recommendations. Based on the teleconsultations, home visits by these nurses were scheduled. There was no specific operational model, as this was something that emerged during the pandemic.

*(…) a lot of technological progress, it’s true that technologies have advanced, information and communication technologies have progressed with this thing of the pandemic and this may be one of the positive aspects. Teleconsultations…*

(28_NSD_49_W)

*We had the advantage of doing quite a lot of telework, in my case that I work in several centers for example…*

(7_SSD_58_M)

#### 3.4.2. Institutional Relations and Social Recognition

The professional performance of CMNs has been valued at the institutional and social levels; some recognition is noticed.

*With this issue of the pandemic it was bad for us on the one hand and very good on the other, because our work has finally been valued*

(1_SSD_62_W)

*It looks as if people respect us a little bit more now. We’ve shown that we’re efficient and that the system needs us*

(24_AD_62_W)

*(…) both older adults and the boards and other institutions like convents have acknowledged this to us, also people belonging to different age groups… everything that came up, hotels for the homeless, hotels for refugees, immigrants…*

(2_SSD_58_W)

#### 3.4.3. Comradeship

CMNs have improved their relations at the level of social-health centers, in addition to specific services such as epidemiology or inspections, among others.

*The connections to other sectors with which we didn’t get so much in contact were strengthened for sure, with epidemiology, with other units, inspections in nursing homes*

(11_SD_50_W)

*(…) the relationship with all the nursing homes was improved, now I know them like the back of my hand*

(6_SSD_57_W)

An important aspect has been preserving comradeship during the pandemic, both among them and with other professional categories.

*To help my peers, who really had a lot of nursing homes, I volunteered to lend a hand and I‘ve had seven nursing homes and three day units to serve besides my job*

(2_SSD_58_W)

*We went there every day, we assembled a medical team from my health center, the three of us went, we weren’t the physician, the nurse or the CMN, no, we were three people from the health center that got in there to help*

(17_SD_55_W)

#### 3.4.4. Burnout Syndrome

Positive and negative feelings were classified into different categories such as positive satisfaction, negative satisfaction, and feelings of satisfaction. Some CMNs mentioned that they are still affected by the situation of work-related stress and work overload for such an extended period of time. Given the number of verbatim testimonies related to this topic, the possibility of a new category emerging from the results can be contemplated, which would be burnout syndrome in some professionals as a consequence of the pandemic.

*I‘ve been really afraid and then the carry-over with this saturation, with the nursing homes… I ended up burned out, I‘ve recovered physically and psychologically with some wear out*

(14_SD_56_W)

*I haven’t gotten over it yet… it’s like when you fracture a bone and they say that it hurts when the climate changes… well I still have it there…*

(17_SD_55_W)

*It was awful for me and I have to tell you that I‘m still somewhat affected with this…*

(3_SSD_62_W)

*The experience from my case management years in the middle of the COVID-19 pandemic is one of the worst nightmares that you might come across in life*

(8_SSD_58_M)

Some CMNs also note that the pandemic affected them both at the professional and personal level, even their family life.

Among these concerns, we can highlight categories (feelings and negative satisfaction) that include negative feelings such as: fear, anguish, dissatisfaction, and difficulties in family tasks and self-care, highlighting the risk of contagion suffered by these professionals.

*(…) my personal life was quite affected, my family life, my three children, well they used to tell me: “But today too, mum?”. Easter, Christmas, 2020 was a bad year… due to the consequences of the pandemic*

(6_SSD_57_W)

## 4. Discussion

The International Council of Nurses and the World Health Organization have raised the need for nurses as a priority. The Nursing Now campaign, in 2020, aimed to implement activities worldwide, promoting professional development, the participation of nurses in health policies, and nurses as leadership figures, something that has been demonstrated in the pandemic. Rojas et al. (2020) agree with the data of the study, in relation to the satisfaction of nurses in the performance of their functions during the pandemic and a greater union in the health teams, better communication, greater visibility, and professional empowerment [9].

Nurses have shown capabilities and potentialities in the face of an international health emergency; for this reason, nursing professionals’ working conditions should be improved throughout the world. Research and education will be fundamental to set forth new initiatives and consolidate the social recognition of this profession [21].

Schiavone and Ferreti (2021) agree that health systems should be improved given the possibility of new pandemics. These authors mention that the COVID-19 pandemic has put health systems around the world to the test, evidencing weaknesses; for this reason, health policies must be changed to enable greater efficacy, focusing more on prevention and fostering new technologies [22].

In Spain, during the COVID-19 pandemic, nurses’ work in primary health care has been very important in relation to detecting cases and fostering phone-based care and video consultations; as reflected in the interviews, there was a technological breakthrough in improving communication and accessibility. The nurses noticed greater appreciation at the social level and have made key interventions in social-health care. At the team level, the pandemic improved unity and communication, in addition to conferring more visibility and empowerment [22,23].

Nursing professionals have made direct interventions, easing sensitization and attitudes towards preventing infections [24].

CMNs adapted well to this new circumstance and showed their commitment and responsibility; as indicated by Chang and Eun (2022), nursing professionals built up their resilience to be able to better adapt to the extremely complex situation imposed by the COVID-19 pandemic [25].

In other regions, Martínez (2020) comments that in the Basque Country, the Department of Health of the Basque Government contracted CMNs during the pandemic, with the aim of establishing a system for early detection; follow-up of cases and their close contacts, from a clinical and epidemiological point of view; and monitoring of the pandemic; which allowed better control during the pandemic [26].

During the pandemic, CMNs increased phone-based care, facing new challenges and role changes while working in the front line against COVID-19 [27].

The pandemic has brought about economic, social, and legal consequences. The professionals faced increased workloads, fear of contagion, and high stress levels due to work-related pressures, exposure to suffering and death, and a shortage of both protective devices and professionals. Nurses have played a fundamental role during the pandemic, ensuring care continuity and showing clinical leadership [28,29].

CMNs devote themselves to a less visible professional activity that includes evaluation, planning, implementation, coordination, and assessment of options and services, which entails significant emotional work. More interdisciplinarity, counselling, and interinstitutional coordination is required to offer a better response to vulnerability [30].

In order to provide good quality care, those in charge of the political decisions should adopt measures to reduce the psychosocial burden borne by nurses during the COVID-19 pandemic [31,32].

Some CMNs expressed negative feelings that might be related to the burnout syndrome; however, others identified very positive outcomes such as resources to deal with the challenges and positive emotional consequences. Advanced nursing practice is required to face pandemic situations [33].

This concept of possible burnout syndrome cases refers to prolonged exposure to the stress generated by the work environment. The at-risk population vulnerable to suffering from the burnout syndrome includes the professionals that work in direct and prolonged contact with the patients [30,31], as well as those committed to their job and those with expectations about their professional goals [32,33].

This important work of the CMNs has been made visible and valued, as stated in the Care Strategy (2020), which comments on the work carried out by the CMNs of the South Health Management area of Seville who have provided significant care during the pandemic—as a reference team for monitoring, prevention, and support—to people living in residential centers and the vulnerable people at home, seven days a week on an uninterrupted basis, performing a daily proactive monitoring and demonstrating the ability to lead highly complex situations, as was done during the pandemic [34].

After the pandemic, CMNs have become a reference figure in primary care in Spain, since the planning and coordination of the most complex and vulnerable people is fundamental; this is consistent with the belief of Pastor et al. (2022) that organizations and institutions should recognize the work of these professionals, given the current scenario of precariousness of the social and health context for the elderly at risk, which has increased during the pandemic. For all of the above, greater interdisciplinarity, counseling, and inter-institutional coordination are needed to provide a better response to vulnerability and avoid referrals to the public prosecutor’s office [25].

Regarding the limitations of this research, we should mention that it has focused on the province of Seville. The CMNs received similar indications throughout the autonomous community, maintaining the same functions, and the work activity was similar. The population served was the same and the competencies and portfolio of services were the same, so these data can easily be extrapolated to other regions; this was the case in Basque Country, where the figure of CMNs achieved positive results in care during the COVID-19 pandemic. In the autonomous community of Madrid, the CMNs received an award for services provided during the pandemic to the most vulnerable groups, including the elderly living in nursing homes [35]. At the international level, the importance of the actions of advanced practice nurses in responding to the health care demand during the COVID-19 pandemic, to ensure the safety and quality of care, was highlighted [36]. 

## Figures and Tables

**Table 1:** Primary health care districts in Seville, frequency of case-management nurses, and frequency of the sample.

Primary Health Care Districts in the Province of Seville	Abbreviations Districts	Frequency of Case-Management Nurses	Frequency of the Sample
Eastern Seville	ESD	5	4
Southern Seville	SSD	13	7
Seville	SD	25	13
Aljarafe	AD	9	4
Northern Seville	NSD	9	3

**Table 2:** Categories, subcategories, and frequency of the verbatim testimonies.

Categories	Subcategories	Frequency of the Verbatim Testimonies
Case-management nurses’ competencies	Workload	13
	Nursing homes	20
	Changes in the competencies	43
Consequences of the COVID-19 pandemic	Technological progress	10
	Institutional relations and social recognition	12
	Comradeship	19
	Burnout syndrome	20

## Data Availability

The original contributions presented in the study are included in the article, further inquiries can be directed to the corresponding author.

## Public Involvement Statement

No public involvement in any aspect of this research.

## Guidelines and Standards Statement

The study follows the Standards for Reporting Qualitative Research (SRQR).
